# Sprouty2 and ‐4 hypomorphism promotes neuronal survival and astrocytosis in a mouse model of kainic acid induced neuronal damage

**DOI:** 10.1002/hipo.22549

**Published:** 2015-11-27

**Authors:** Sitthisak Thongrong, Barbara Hausott, Letizia Marvaldi, Alexandra S. Agostinho, Luca Zangrandi, Johannes Burtscher, Barbara Fogli, Christoph Schwarzer, Lars Klimaschewski

**Affiliations:** ^1^ Division of Neuroanatomy, Department of Anatomy Histology and EmbryologyMedical UniversityInnsbruck6020InnsbruckAustria; ^2^ Department of PharmacologyMedical University InnsbruckInnsbruck6020Austria

**Keywords:** hippocampus, gliosis, ERK, Sprouty, neurodegeneration, granule cell dispersion

## Abstract

Sprouty (Spry) proteins play a key role as negative feedback inhibitors of the Ras/Raf/MAPK/ERK pathway downstream of various receptor tyrosine kinases. Among the four Sprouty isoforms, Spry2 and Spry4 are expressed in the hippocampus. In this study, possible effects of Spry2 and Spry4 hypomorphism on neurodegeneration and seizure thresholds in a mouse model of epileptogenesis was analyzed. The Spry2/4 hypomorphs exhibited stronger ERK activation which was limited to the CA3 pyramidal cell layer and to the hilar region. The seizure threshold of Spry2/4^+/−^ mice was significantly reduced at naive state but no difference to wildtype mice was observed 1 month following KA treatment. Histomorphological analysis revealed that dentate granule cell dispersion (GCD) was diminished in Spry2/4^+/−^ mice in the subchronic phase after KA injection. Neuronal degeneration was reduced in CA1 and CA3 principal neuron layers as well as in scattered neurons of the contralateral CA1 and hilar regions. Moreover, Spry2/4 reduction resulted in enhanced survival of somatostatin and neuropeptide Y expressing interneurons. GFAP staining intensity and number of reactive astrocytes markedly increased in lesioned areas of Spry2/4^+/−^ mice as compared with wildtype mice. Taken together, although the seizure threshold is reduced in naive Spry2/4^+/−^ mice, neurodegeneration and GCD is mitigated following KA induced hippocampal lesions, identifying Spry proteins as possible pharmacological targets in brain injuries resulting in neurodegeneration. The present data are consistent with the established functions of the ERK pathway in astrocyte proliferation as well as protection from neuronal cell death and suggest a novel role of Spry proteins in the migration of differentiated neurons. © 2015 The Authors Hippocampus Published by Wiley Periodicals, Inc.

## INTRODUCTION

Mesial temporal lobe epilepsy is one of the most common types of epilepsy characterized by recurrent spontaneous seizures that often result in hippocampal sclerosis and granule cell dispersion (GCD). Several antiepileptic drugs are available, however, one third of patients remain refractory to pharmacological treatment (Kwan et al., 2011). Novel anticonvulsant therapies aim at reducing the effects of glutamatergic overflow and promoting inhibitory activities of the GABAergic system. Seizure induced excitotoxicity results in neurodegeneration by activation of intraneuronal signaling pathways including the MAPK subfamilies ERK1/2, c‐Jun amino‐terminal kinases (JNK), and p38 kinases (McNamara et al., 2006).

Activated extracellular signal‐regulated kinase (ERK) regulates key aspects of cellular proliferation, differentiation, and survival (Marshall, 1995; Ballif and Blenis, 2001; Cheng et al., 2013). Phosphorylated ERK1/2 (pERK) was long thought to promote cell survival in the adult nervous system, whereas JNK and p38 MAPK were shown to be involved in cell death. However, several studies suggested a role for pERK in promotion of cell death as well. It is now generally assumed that the temporal changes in ERK activation as well as its intracellular localization determine cell fate decisions such as survival, death or proliferation. Nuclear translocation of active ERK in post‐mitotic neurons appears to promote cell death, whereas ERK activation in the cytoplasm results in neuronal survival (reviewed in Subramaniam and Unsicker, 2010).

Intracellular negative feedback inhibitors of receptor tyrosine kinase (RTK) signaling such as the Sprouty (Spry) proteins play a key role in ERK regulation (Mason et al., 2006). They are induced by RTK activation and function as growth factor antagonists by specific interference mainly with processes upstream of ERK (Hanafusa et al., 2002; Wong et al., 2002; Sasaki et al., 2003; Ozaki et al., 2005). Spry1, ‐2, and ‐4 represent the major isoforms in the brain, whereas Spry3 is detected at low levels only (Minowada et al., 1999). Spry1 and ‐2 are strongly expressed in early neural plate stages and involved in cortical proliferation and differentiation (Faedo et al., 2010). Although they progressively decrease during differentiation and postnatal development, Spry2 and ‐4 persist in the adult cortex and hippocampus. Spry2/4 are ubiquitiously expressed in pyramidal cells, interneurons, and glial cells of the hippocampus, and their reduction promotes neuronal survival (Gross et al., 2007) as well as axon regeneration in vitro and in vivo (Hausott et al., 2012; Marvaldi et al., 2015).

The aim of this study was to investigate whether Spry2/4 hypomorphism (global double‐knockout mice are not viable) would influence ERK1/2 activation and neuronal survival in the kainic acid (KA) local injection model of temporal lobe epileptogenesis (Magloczky and Freund, 1993; Loacker et al., 2007). For this purpose, we compared naive, saline, and KA injected animals by means of histomorphological, neurochemical, and behavioral analysis.

## MATERIALS AND METHODS

### Animals

Germline Spry2 and Spry4 knockout mice were obtained from MMRRC (Mutant Mouse Regional Resource Centre, University of North Carolina at Chapel Hill) on a mixed genetic background. Standard back‐crossing was performed on a 129 S1/SVImJ background until the 10th generation. Heterozygous double Spry2/4 knockout mice (Spry2/4^+/−^) were generated by crossing Spry2^+/−^ and Spry4^+/−^ mice of either sex. They were heavier than wildtype (WT) mice, but no other obvious differences in fertility or behavior were observed. All in vivo experiments were performed with the approval of the ethical commission of the Austrian government (BMWF‐66.011/0184‐II/3b/2011). The guidelines of the EU (Directive 2010/63/EU) concerning animal care were closely observed, and every effort was taken to minimize the number of animals used.

### Quantitative RT‐PCR

Naive WT and Spry2/4^+/−^ mice were sacrificed by cervical dislocation. The hippocampi were dissected, immediately frozen in liquid nitrogen, and used for RNA extraction with TriReagent^®^ RT (Molecular Research Center) according to the manufacturer's instructions. Reverse transcription was performed with 1 µg RNA in a total volume of 20 µL using the iScript^TM^ cDNA Synthesis Kit (Biorad). Quantitative real‐time PCR was performed on Biorad's iCycler in a final volume of 12.5 µL iQ SYBR Green Supermix (Biorad), 7.5 µL sterile water, 2 µL of each primer (160 nM), and 1 µL cDNA template. The reactions were carried out in 40 cycles (3 minutes denaturing step followed by 40 cycles 1 minute at 94°C, 30 seconds at 60°C, and 1 minute at 72°C). We applied primers for HPRT1 as control (sense 5′dTGACACTGGCAAAACAATGCA, antisense 5′dGGTCCTTTTCACCAGCAAGCT), Spry2 (sense 5′dCACGGAGTTCAGATGTGTTCTAAGC, antisense 5′dATGTTTGTGCTGAGTGGAGGGG) and Spry4 (sense 5′dTCGGGTTCGGGGATTTACAC, antisense 5′dGGCTGGTCTTCATCTGGTCAATG).

### Seizure Threshold

Seizure threshold was determined by pentylenetetrazole (PTZ) tail‐vein infusion on freely moving mice at a rate of 100 µL/min (10 mg/mL PTZ in saline, pH 7.4) before and 4 weeks after KA injection. The threshold dose was calculated from the infusion volume in relation to the body weight. Infusion was stopped when the mice displayed generalized clonic seizures (Loacker et al., 2007).

### Intrahippocampal Injections of KA

To study neuronal degeneration and hippocampal reorganization, KA was injected into the dorsal hippocampus of deeply anesthetized male mice 12–16 weeks of age (ketamine 160 mg/kg i.p. followed by sevoflurane through a precise vaporizer). They were immobilized using a stereotactic apparatus (David Kopf, Bilaney) with the nose bar adjusted to equal heights of lambda and bregma reference points. Coordinates from bregma (1.8 mm posterior, 1.8 mm lateral, and 1.7 mm below the skull) were chosen to target the stratum radiatum of CA1 in the left dorsal hippocampus (Paxinos and Franklin, 2001). KA (1 nmol in 50 nL saline, pH 7.2) was applied with a 0.5 µL Hamilton syringe over a period of 2 minutes with the canula kept in place for 5 minutes followed by stepwise retraction (0.3 mm/min) to minimize backflux. Three weeks later mice were either subjected to behavioral testing or killed by an overdose of thiopental (150 mg/kg), and brains were fixed by transcardial perfusion with 4% paraformaldehyde in PBS (50 mM phosphate buffered saline, pH 7.2). All animals received metoxicam (2 mg/kg) 30 minutes before and 12 h after surgery as analgesic treatment. Naive animals and age‐matched saline injected mice were used as controls. In response to KA injection, all mice showed status epilepticus followed by spontaneous recurrent seizures developing within about 2 weeks.

### Histological Analysis

Immunohistochemistry and Nissl (cresyl violet) staining were performed on coronal 30 µm vibratome sections incubated free‐floating in blocking solution (10% normal goat serum and 0.3% Triton X‐100 in TBS) for 90 minutes. Primary antibodies against prosomatostatin (STT, 1:2,000, Novusbio), neuropeptide Y (NPY, 1:500, ImmunoStar), pERK (Cell Signaling, 1:250), ERK (Cell Signaling, 1:250), Reelin (Chemicon, 1:1,000), or glial fibrillary acidic protein (GFAP, 1:10, ImmunoStar) were applied overnight at RT followed by horseradish peroxidase conjugated secondary antibodies (1:500, Dako) and 3,3′‐diaminobenzidine for detection (constant incubation times in DAB for all groups). For pERK/ERK staining fluorescently labeled secondary antibodies (Alexa‐488 goat anti‐rabbit, Alexa‐594 chicken anti‐mouse, 1:1,000, Invitrogen) were applied for 30 minutes at RT.

**Figure 1:**
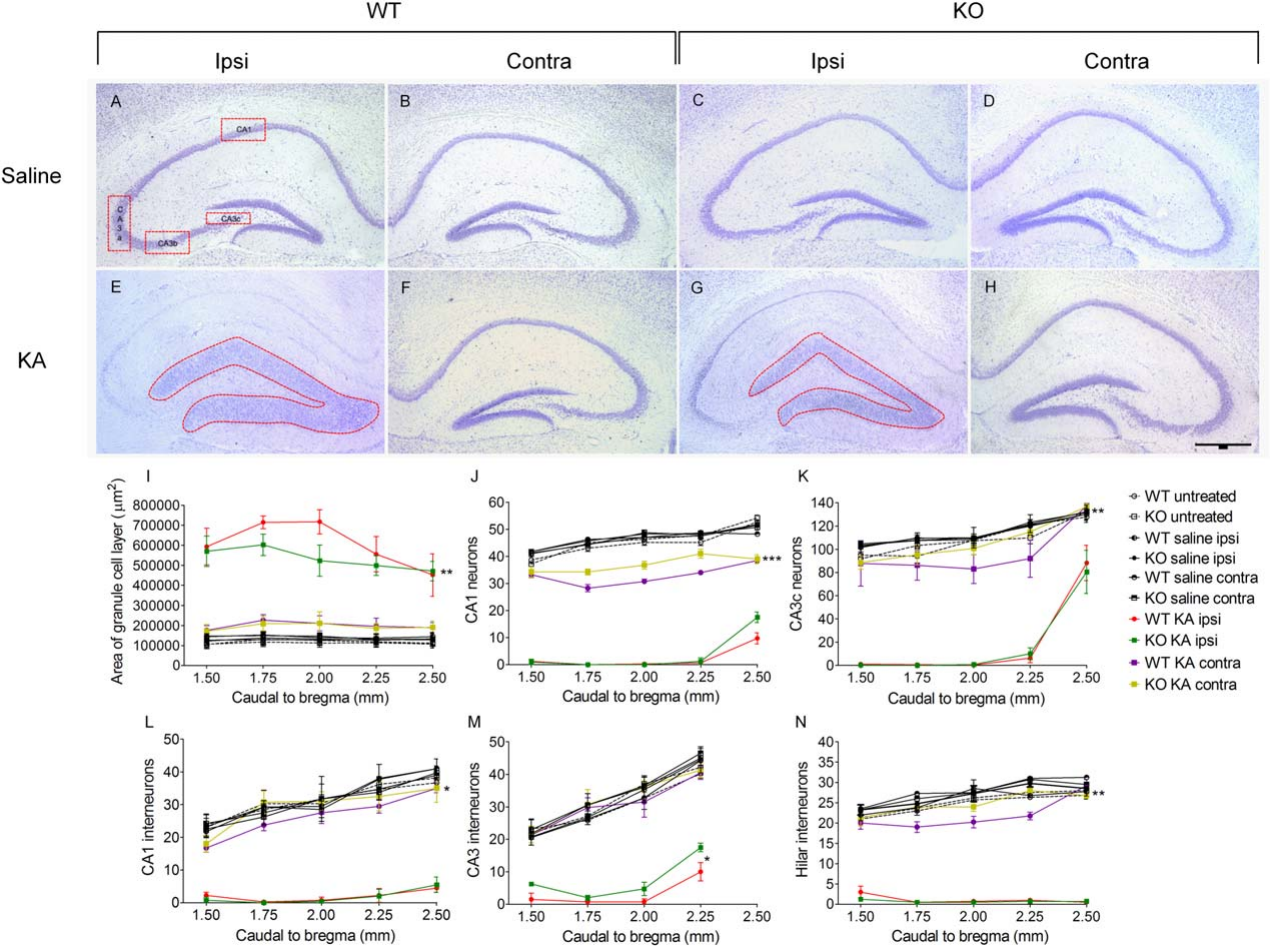
Nissl staining followed by stereological analysis of neuronal loss and GCD 3 weeks after unilateral intrahippocampal injection of saline or KA. Images of 30 µm sections of the dorsal hippocampus near the injection site are shown (1.8 mm caudal to bregma). KA induced cell death and GCD is clearly visible in WT (**A, B, E, F**) and in Spry2/4^+/−^ mice (**C, D, G, H**). *Red boxes* in panel A indicate the hippocampal subregions analyzed in this study. Quantifications reveal alterations in the total area of the granule cell layer along the rostro‐caudal axis of the dorsal hippocampus (**I**) and distribution of CA1 and CA3c principal neurons (**J, K**) as well as interneurons of the CA1, CA3, and hilar region (**L–N**). Mean ± SEM (*n* = 4), one‐way ANOVA of “area under the curve” (AUC), bar = 500 µm. [Color figure can be viewed in the online issue, which is available at wileyonlinelibrary.com.]

Cell counts and morphometric measurements of the granule cell layer (GCL) were performed on every sixth section of the dorsal hippocampus (at 1.3–2.5 mm from bregma) obtained from four experimental animals per group (values from both hippocampi were averaged for naive untreated mice). Due to KA diffusion the focal lesion extended along the rostro‐caudal axis. Therefore, five cell counts were performed at different locations covering at least 500 µm rostral and caudal of the injection. Data points were integrated for the calculation of the “area under the curve” followed by statistical comparison. Interneurons were counted in the entire hippocampal subfields of the hilus, CA1, and CA3. Pyramidal neurons were assessed in regions of interest (125 µm length, covering the whole width of the layer) as indicated in Figure 1A.

Reactive astrocytes were detected by GFAP labeling and analyzed in sections nearby the injection site (1.6–1.8 mm behind bregma). The average GFAP staining intensity of reactive astrocytes in the area of the cortex and molecular layer of the dentate gyrus was analyzed by MetaMorph^®^ software (Visitron Systems).

### Western Blots

Dorsal hippocampi taken from animals three days after KA injection or from untreated mice were homogenized in 70 µL lysis buffer (50 mM Tris/HCl pH 7.4, 500 mM NaCl, 1% NP‐40, 0.5% Na‐desoxycholate, 0.1% SDS, 0.05% NaN3) supplemented with 20 µg/mL complete protease inhibitor cocktail (Roche Diagnostics) and phosphatase inhibitor cocktail II and III (1:100, Sigma) using the Microson Ultrasonic Cell Disruptor. Lysates were centrifuged for 20 minutes at 4°C and 10,000*g*. Protein concentrations of the supernatants were determined using Bradford protein assay reagent (Biorad) and aliquots containing 20 µg of protein were analyzed by electrophoresis on SDS‐polyacrylamide gels and then transferred to PVDF‐FL membranes. After primary antibody incubation (pERK antibodies from Cell Signaling 1:1,000 or Abcam 1:10,000; ERK antibodies from Cell Signaling 1:2,000 or Abcam 1:1,000) the secondary fluorescent‐linked antibodies (IRDye 680RD goat anti‐mouse and IRDye 800CW goat anti‐rabbit, LI‐COR Biosciences, 1:20,000) were detected by the Odyssey Infrared Imaging System (LI‐COR Biosciences) and quantified using Image Studio Lite 5.2 Software.

### Statistical Analysis

Statistical analysis was performed using ANOVA followed by Bonferroni's multiple comparison test applying Prism software (* = *P* < 0.05, ** = *P* < 0.01, *** = *P* < 0.001).

## RESULTS

### Regulation of Spry2/4 mRNA in Response to Excitotoxic Lesion

Quantitative RT‐PCR revealed that in dorsal hippocampi of Spry2/4^+/−^ mice Spry2 mRNA levels significantly decreased by 72 (±9)% and Spry4 mRNA by 49 (±10)% in comparison to WT mice (*n* = 3, *P* < 0.05). Three days following KA injection, Spry2 mRNA significantly increased 2.8 fold (±0.5, *n* = 3, *P* < 0.05) in the dorsal hippocampus of WT animals, whereas Spry4 mRNA was elevated by a factor of 2 (±0.4, n.s.). In contrast, no up‐regulation was detected in Spry2/4^+/−^ mice (Spry2 mRNA changed 1.6 ± 0.2 fold, Spry4 mRNA 1.1 ± 0.3 fold) and Spry2/4 mRNA levels were not regulated in the contralateral hippocampus after lesion either.

### Changes in Seizure Threshold in Spry2/4^+/−^ Mice

To determine possible differences in seizure susceptibility between WT and KO mice, the pentylenetetrazole (PTZ) tail‐vein infusion test was performed in naïve and KA treated animals. Whereas the Spry2/4 hypomorphs at naive state exhibited a seizure threshold of 36.6 ± 2.3 mg PTZ/kg (10 mg/mL; 100 µL/min via tail‐vein infusion), higher PTZ concentrations were required to induce clonic seizures in WT mice (41.1 ± 2.2 mg PTZ/kg body weight). This was not surprising, because ERK activation had been demonstrated to cause epilepsy by stimulating NMDA receptor activity (Nateri et al., 2007). However, the repetition of the PTZ experiment in these groups 1 month after KA injection into the stratum radiatum of the dorsal hippocampus (CA1) revealed a reduction of seizure thresholds to similar values in both groups (31.0 ± 3.9 mg for Spry2/4^+/−^ mice and 32.4 ± 2.9 mg PTZ/kg for WT).

### Histological Analysis after KA Injection

KA injection produced status epilepticus lasting for about 48 hr in all treated animals. Following this initial status, mice developed self sustained focal and generalized seizures within several days as described before (Loacker et al., 2007). Morphological differences became clearly apparent in the subchronic phase (3 weeks after injury). Particularly, a broadening of the normally compact granule cell layer along the rosto‐caudal axis was observed (Figs. 1A,E and C,G). This GCD was significantly less pronounced in Spry2/4^+/−^ as compared with WT mice (536,623 ± 23,435 µm^2^ vs. 627,500 ± 17,751 µm^2^ in WT; *P* < 0.01; Fig. 1I). GCD was not detected in the contralateral hippocampus of KA injected or in saline treated mice. Untreated WT and Spry2/4^+/−^ mice did not show obvious differences with regard to overall cortical, hippocampal, striatal, or thalamic anatomy in coronal Nissl stained brain sections.

Overall neuron number was determined by counting cellular profiles in every sixth Nissl stained section. Principal neurons of CA1 and all pyramidal neurons of the CA3c subfield were assessed along the rostro‐caudal axis over 1 mm (injection site at 1.8 mm caudal to bregma). Interneurons were counted in the entire hippocampal subfields of the hilus, CA1, and CA3. For statistical analysis the “areas under the curve” were compared. Degeneration of principal neurons was prominent in CA1 and CA3c of the ipsilateral and, to a lesser degree, also of the contralateral dorsal hippocampus following KA (Figs. 1E–H). Noteworthy, more principal neurons were detected contralaterally in CA1 of Spry2/4^+/−^ mice (37 ± 1.3 vs. 32 ± 0.05 in WT; *P* < 0.001; Fig. 1J) and in CA3c (106 ± 2.1 vs. 94 ± 4.2 in WT; *P* < 0.01; Fig. 1K). Scattered neurons were protected by Spry2/4 hypomorphism contralaterally in CA1 (30 ± 1.1 vs. 27 ± 0.9 in WT; *P* < 0.05; Fig. 1L), ipsilaterally in CA3 (5 ± 0.4 vs. 2 ± 0.1 in WT; *P* < 0.05; Fig. 1M), and contralaterally in the hilar region (25 ± 0.5 vs. 21 ± 0.8 in WT; *P* < 0.01; Fig. 1N).

### Alterations in Reelin Expression

Since KA induced GCD was suggested to be linked to decreased expression of Reelin (Haas et al., 2002), an extracellular matrix protein released by hilar interneurons, we determined the number of Reelin‐immunoreactive cells. Although Reelin positive neurons were reduced by greater than 80% following KA injection, no significant differences were observed in Spry2/4^+/−^ mice as compared with WT in the ipsilateral CA1 area (5 ± 2.5 vs. 4 ± 2.3 in WT), in CA3 (5 ± 2.0 vs. 4 ± 2.0 in WT), in the molecular layer (11 ± 0.5 vs. 10 ± 1.0 in WT) or in the hilus (1 ± 0.08 vs. 1 ± 0.2 in WT). The contralateral hippocampus of Spry2/4^+/−^ mice displayed only a slight reduction in Reelin labeled cells after KA treatment in CA1 (31 ± 3.6 vs. 29 ± 3.9 in WT), in CA3 (23 ± 3.7 vs. 22 ± 2.7 in WT), in the molecular layer (16 ± 0.7 vs. 17 ± 0.8 in WT), or in the hilus (21 ± 1.0 vs. 20 ± 1.0 in WT) with no significant changes between the two genotypes.

### Alterations in Neuropeptide Expression

**Figure 2:**
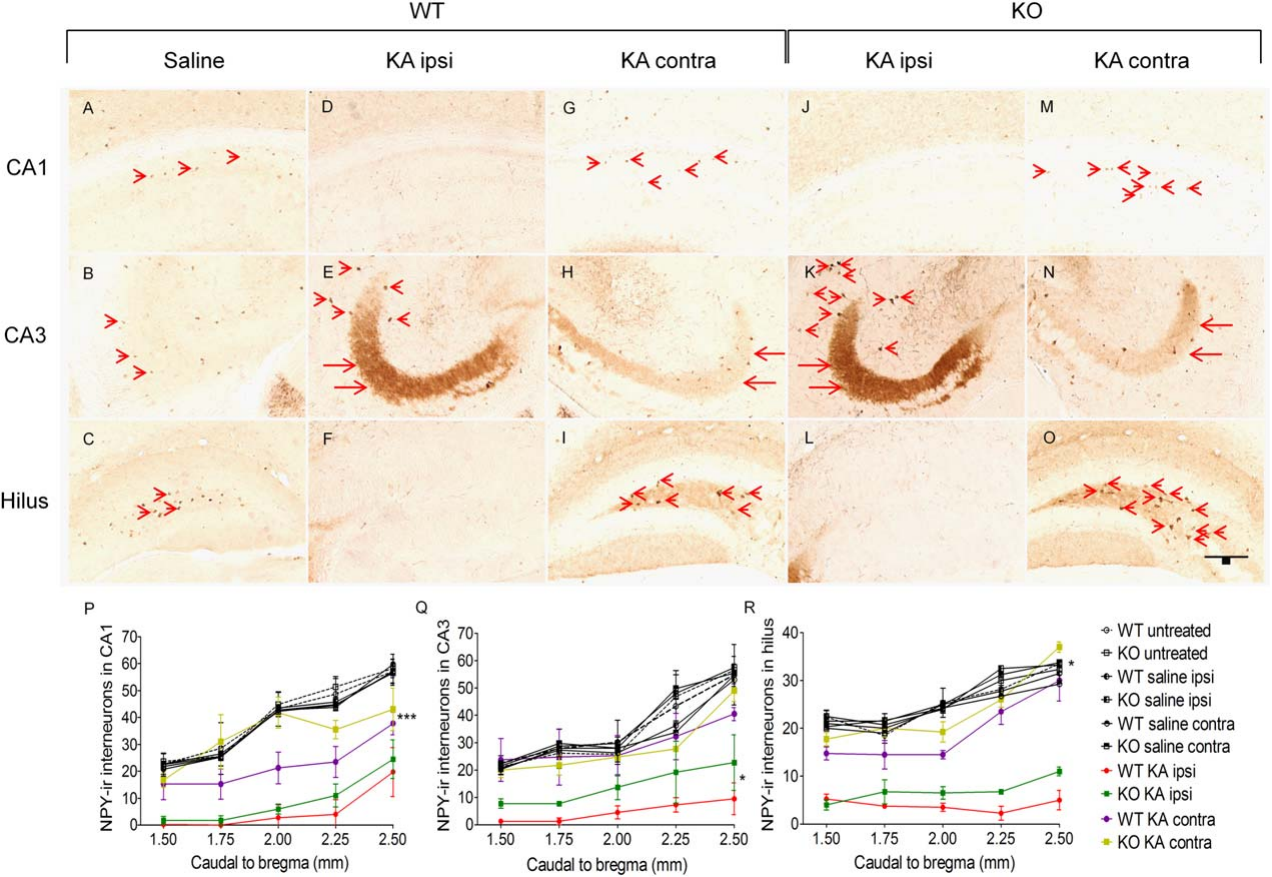
Neuropeptide Y (NPY) immunoreactivity 3 weeks after unilateral injection of saline or KA into the dorsal hippocampus (*arrows* in E, H, K, and N indicate NPY positive mossy fibers). NPY immunoreactive interneurons (*arrowheads*) are lost in the ipsilateral hippocampus of CA1 (**A–M, P**) and hilus (**C–O, R**) of both groups, whereas NPY positive cells in CA3 (**B–N, Q**) are mostly conserved in Spry2/4^+/−^ mice. Note that in the contralateral hippocampi CA1 and hilar interneurons are mostly spared in Spry2/4^+/−^ hypomorphs. No differences in numbers of NPY positive interneurons are detected following saline injection. Mean ± SEM (*n* = 4), one‐way ANOVA, bar = 100 µm. [Color figure can be viewed in the online issue, which is available at wileyonlinelibrary.com.]

The expression of neuropeptide Y (NPY) in hippocampal granule cells and interneurons is activated by the release of glutamate during seizures, and NPY positive interneurons frequently co‐express somatostatin (SST). As shown in earlier studies (Gruber et al. 1994; Schwarzer and Sperk, 1998), NPY is observed in mossy fibers of the hippocampus in KA injected animals indicating spontaneous seizure activity (Figs. 2E,K). The numbers of NPY positive interneurons in ipsilateral areas CA1, CA3 and in the hilus were markedly reduced after KA injection (Figs. 2A–F and J–L). Spry2/4^+/−^ mice displayed protection of NPY interneurons contralaterally in CA1 (35 ± 2.2 vs. 22 ± 2.6 in WT; *P* < 0.001; Fig. 2P), ipsilaterally in CA3 (14 ± 2.4 in KO vs. 5 ± 0.7 in WT; *P* < 0.05; Fig. 2Q) and contralaterally in the hilus (24 ± 3.5 vs. 20 ± 3.2 in WT; *P* < 0.05; Fig. 2R) as compared with WT mice after KA treatment. No difference was detected in NPY expression between the genotypes following saline injection or in naive mice.

SST is expressed in GABAergic interneurons that are found in hippocampal areas CA1, CA3, and in the hilus (Kosaka et al., 1988) and highly vulnerable to KA induced cell death (Magloczky and Freund, 1993). In this study, naive and saline injected mice revealed no significant differences in number of STT positive neurons in the hippocampal areas CA1 and CA3 or in the hilar region. Three weeks after KA injection, however, all groups displayed severe reductions in SST immunoreactive neurons primarily in the ipsilateral hippocampus. WT mice exhibited significantly lower numbers of SST positive neurons as compared with Spry2/4^+/−^ mice in the CA1 region (ipsilaterally 12 ± 0.8 vs. 8 ± 1.0 in WT; *P* < 0.05 and contralaterally 30 ± 1.4 vs. 24 ± 0.5 in WT; *P* < 0.001). In area CA3 significantly more SST expressing neurons were observed in Spry2/4^+/−^ mice ipsilaterally (18 ± 0.6 vs. 13 ± 0.9 in WT; *P* < 0.001), whereas the hilar region revealed more SST positive neurons in Spry2/4^+/−^ mice contralateral to the injection site (20 ± 0.2 vs. 17 ± 1.2 in WT; *P* < 0.05).

### Alterations in Glial Fibrillary Acidic Protein

**Figure 3:**
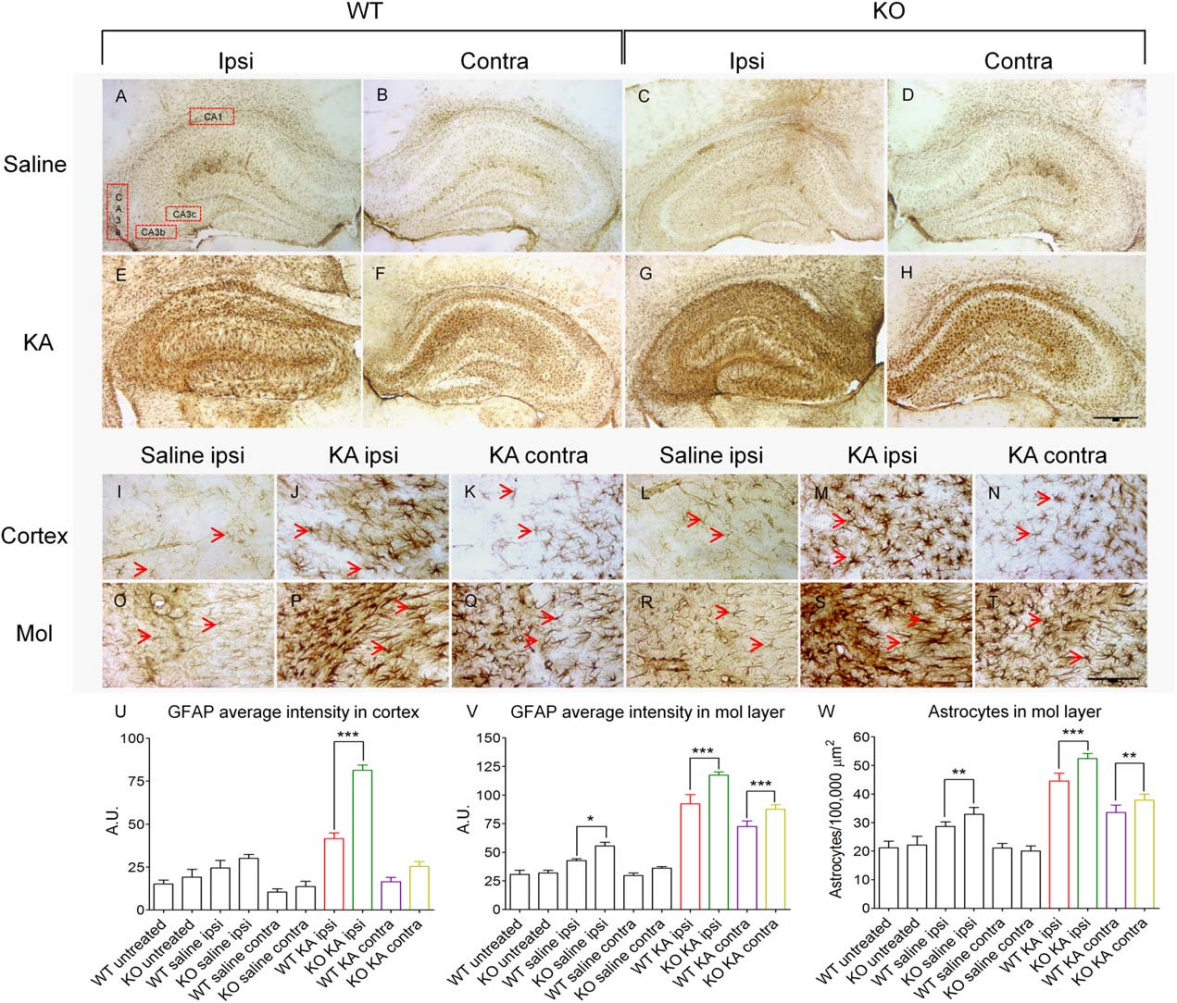
Glial fibrillary acidic protein (GFAP) staining 3 weeks after unilateral injection of saline or KA into the dorsal hippocampus near the injection site (1.8 mm caudal to bregma). As compared with saline injection (**A–D**), prominent KA induced reactive astrocytosis is detected in the ipsilateral and contralateral hippocampus of both genotypes (**E–H**). Spry2/4^+/−^ mice reveal higher GFAP intensity in the ipsilateral and contralateral molecular layers as compared with WT mice. Higher magnification of the cortex (**I–N**) and the molecular layer (Mol, **O–T**) are shown to reveal single astrocytes and their processes (*arrowheads*). Quantifications of GFAP average staining intensity in the cortex (U) and of the molecular layer (V) correlate with increased numbers of reactive astrocytes (W). Mean ± SEM (*n* = 4), one‐way ANOVA, bar = 500 µm (**A–H**) or 100 µm (**I–T**). Colored bar labeling corresponds to legend in Figures 1 and 2. [Color figure can be viewed in the online issue, which is available at wileyonlinelibrary.com.]

Reactive astrocytosis is increased in response to injury, inflammation or epilepsy (Devinsky et al., 2013). The process begins almost immediately after injury and has beneficial functions to limit damage through glial scar formation and, thereby, promote recovery. Three weeks following KA injection GFAP labeling was increased in both hemispheres as indicated by the increased size and number of astrocytic processes in the hippocampus (Figs. 3A–H) and in other areas (Figs. 3I–T). The average staining intensity of GFAP in the cortex around the injection site was markedly enhanced in Spry2/4^+/−^ mice as compared with WT (81.4 ± 3.1 arbitrary units [a.u.] in KO vs. 41.5 ± 3.4 a.u. in WT; *P* < 0.001; Fig. 3U). GFAP labeling within the dispersed granule cell layer or in the contralateral cortex was not different between the two groups, and in untreated mice no difference was observed either. However, Spry2/4^+/−^ mice exhibited increased GFAP average intensity in the ipsilateral molecular layer of the dentate gyrus following saline injection (55.5 ± 3.2 a.u. vs. 42.7 ± 1.5 a.u. in WT; *P* < 0.001; Fig. 3V).

After KA injection stronger average intensity of GFAP staining was observed in both hippocampi (118 ± 1.0 a.u. in KO vs. 92.2 ± 2.9 a.u. in WT; *P* < 0.001 ipsilaterally and 87.6 ± 4.0 a.u. in KO vs. 72.4 ± 1.8 a.u. in WT; *P* < 0.001 contralaterally). This could be attributed to a difference in glial proliferation, since the molecular layer of the dentate gyrus displayed significant differences in numbers of reactive astrocytes (52.4 ± 0.6/10,000 µm^2^ in KO vs. 44.6 ± 0.9/10,000 µm^2^ in WT; *P* < 0.001 ipsilaterally and 37.9 ± 0.7/10,000 µm^2^ in KO vs. 33.6 ± 0.9/10,000 µm^2^ in WT; *P* < 0.01 contralaterally; Fig. 3W).

### Alterations in ERK Activation

**Figure 4:**
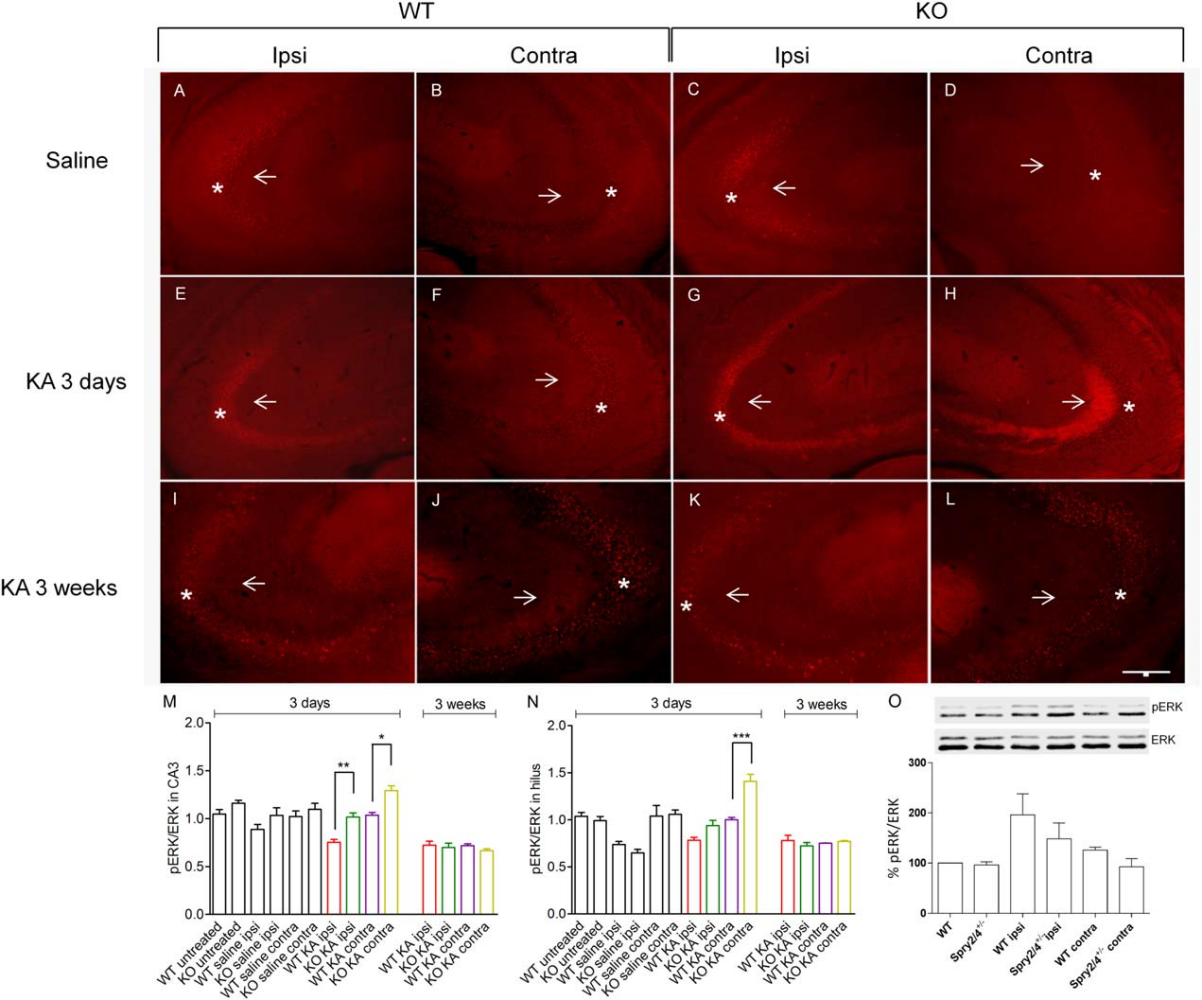
Immunolabeling for pERK in CA3 and in the hilus following unilateral injection of saline (**A–D**) or KA (**E–L**) into the dorsal hippocampus. KA prominently induced increases in axonal pERK labeling in the contralateral stratum lucidum of CA3 (**F, H**; *arrows*) in both genotypes 3 days following the lesion. In the ipsilateral and contralateral pyramidal cell layer (*asterisk*; punctate labeling most likely corresponds to axonal endings) Spry2/4^+/−^ mice exhibit higher pERK staining as compared with WT littermates after KA injection as well (**E, G**). Three weeks after KA treatment weaker labeling intensities and no significant differences between the groups are observed (**I–L**). Quantifications of pERK/ERK average intensity measurements in CA3 and hilus, respectively (**M, N**). Mean ± SEM (*n* = 4), one‐way ANOVA, bar = 200 µm. Colored bar labeling corresponds to legend in Figures 1 and 2. (**O**) shows Western Blot analysis of pERK/ERK activation in the whole dorsal hippocampus 3 days after KA injection compared with naïve control animals (similar results were obtained with antibodies from two different companies). Band intensities were quantified and the ratio of pERK relative to ERK of six independent experiments is shown. [Color figure can be viewed in the online issue, which is available at wileyonlinelibrary.com.]

Sprys function as growth factor antagonists, thereby fine‐tuning RTK activities mainly by specific interference with processes upstream of ERK following RTK stimulation. It was shown previously that pERK increases markedly after the onset of spontaneous or evoked seizures and decreases shortly thereafter (Houser et al., 2008). Therefore, we investigated pERK immunolabeling in WT and Spry2/4^+/−^ mice 3 days after the initial status epilepticus and in the subchronic stage of progressing neuronal damage (21 days after KA injection). Compared with the KA injected brains, the control groups revealed weaker pERK immunofluorescence in the pyramidal cell layer of the hippocampus (CA1–CA3) and in the molecular layer as well as in the mossy fiber projection to the stratum lucidum of CA3 (Figs. 4A–D). Whereas no differences were observed in total ERK immunoreactivity (data not shown), pERK staining intensities in cyto‐ and axoplasm differed between WT and KO mice 3 days following KA treatment, in the ipsi‐ and contralateral stratum lucidum of layer CA3 (Figs. 4E–H, M) and in the contralateral hilar region of the hippocampus (Fig. 4N). As reported before (Houser et al., 2008), pERK/ERK levels decrease in the hippocampus during the chronic period following seizure induction. In untreated and saline injected groups, no differences in ERK activation were detected. Although a trend toward increased pERK/ERK levels was observed 3 days after the lesion in the ipsilateral dorsal hippocampus, Western blot analysis of total hippocampal extracts applying three different pERK antibodies did not reveal statistically significant differences between KO and WT mice (Fig. 4O) suggesting that the changes in ERK activation are subtle and confined to specific hippocampal subregions as revealed by immunofluorescence.

## DISCUSSION

In this animal model of KA induced epileptogenesis, stimulation of kainate receptors results in enhanced glutamate release causing neuronal damage via Ca^2+^ influx through NMDA receptor channels (Fariello et al., 1989). Due to commissural stimulation and generalization of induced seizures, the degenerative changes are not restricted to the injected hippocampus, but are observed in the contralateral hippocampus and other brain areas as well. The appearance of NPY immunoreactivity in mossy fibers of all KA‐treated animals ipsi‐ and contralaterally are suggestive of increased network activity involving both hippocampi (Schunk et al., 2010).

In the present study, it is demonstrated that Spry2 and ‐4 participate in KA induced neurodegeneration possibly through inhibition of ERK signaling, although the contribution of other pathways cannot be ruled out (Edwin et al., 2009). Spry2/4 hypomorphs exhibit reduced neuronal loss bilaterally with regard to principal neurons and interneurons. Moreover, increased astrocytosis is detected in both hippocampi and reduced neuronal migration in the ipsilateral granule cell layer of Spry2/4^+/−^ mice.

Considering the molecular mechanisms of Spry function, it has been shown that Spry2 and ‐4 are induced transcriptionally by growth factor stimulation and translocated from the cytosol to the cell membrane where they become anchored through palmitoylation and phosphorylated at tyrosine residues (Impagnatiello et al., 2001; Mason et al., 2004). Spry2 activation requires phosphorylation at the essential Tyr55 residue. Spry4 is not phosphorylated in response to RTK stimulation, however, the Tyr53 residue is necessary for the inhibitory activity of Spry4 (Alsina et al., 2012). Spry2 and ‐4 then bind the adaptor protein Grb2 to inhibit the recruitment of the Grb2‐SOS‐complex to FRS2 or Shp2, which prevents downstream Ras‐ERK activation (Hanafusa et al., 2002).

In primary neurons and in C6 glioma cells, down‐regulation of Spry2 leads to the activation of Ras and ERK, whereas phosphorylation of Akt and p38 MAPK remains unchanged (Hausott et al., 2009). This results in enhanced axon elongation and promotes neuronal survival (Gross et al., 2007). We observed activated ERK primarily in the cytoplasm, but not in the nucleus of Spry2 deficient peripheral neurons (Hausott et al., 2009; Marvaldi et al., 2015). Although speculative at the moment, this may be caused by different activities of the ERK1/2 inactivating MAP kinase phosphatases (Tsang and Dawid, 2004). Whereas MKP1 is located in the nucleus, MKP3 is a cytoplasmic enzyme.

With regard to the lesion model used here, the strong and sustained activation of nuclear ERK results in neuronal death as observed with glutamate overflow for more than 6 hours (Luo and DeFranco, 2006). Luo and DeFranco also demonstrated that a transient, primarily cytoplasmic ERK1/2 activation reflects a pro‐survival response and showed that chronic ERK1/2 activation itself is not sufficient to induce cellular toxicity, but requires cooperation with other pathways. Nevertheless, several reports over the years convincingly demonstrated that overall inhibition of MEK/ERK by PD98059 or U0126 protects against oxidative stress (reactive oxygen species activate ERK1/2) as well as against nitric oxide, dopamine or β‐amyloid induced neuron death. In contrast, reduction of the negative feedback inhibitors of ERK1/2 signaling, Spry2 and −4, enhances cytosolic/axoplasmic pERK levels and counteracts KA/glutamate dependent neurotoxicity as demonstrated here. This apparent contradiction cannot be resolved at the moment, but different lesion paradigms may be accompanied by different spatiotemporal activation patterns of ERK1/2. Moreover, in addition to Grb2, other putative interaction partners of Sprys have been described that may be involved in the effects observed in the present study, for example, c‐Cbl, caveolin‐1, dual specificity kinase TESK1 and the protein tyrosine phosphatase PTP1B (Mason et al., 2006; Edwin et al., 2009).

The most prominent differences in the particular lesion model used here relate to neuronal survival, GCD, and reactive astrogliosis. It is likely that astrocytes with reduced Spry2/4 levels respond to an insult with stronger proliferation via enhanced ERK signaling. Constitutively active MEK1 results in a dramatic increase in the number of astrocytes (Li et al., 2012), and inhibition of MEK1 with PD98059 blocks astrocyte proliferation (Barbero et al., 2002). In recent years, the prevalent view of reactive astrogliosis as an inhibitor of regeneration has changed into a more optimistic prospect emphasizing the beneficial functions of astrogliosis and scar formation. In fact, reactive astrocytes protect neurons by uptake of excitotoxic glutamate that is impaired in mice deficient of the astrocyte glutamate transporter (Rothstein et al., 1996). Furthermore, astrocytes act as buffers of the extracellular fluid, thereby, normalizing seizure thresholds (Zador et al., 2009). Attenuation of reactive astrogliosis clearly increases neuronal cell death, lesion size and demyelination resulting in a dramatic loss of function (reviewed by Sofroniew, 2009). Furthermore, immunological elimination of astrocytes causes seizures as observed in Rasmussen's disease (Bauer et al., 2007). Taken together, stimulation of reactive astrogliosis in Spry2/4 deficient mice may have stabilized the seizure threshold following KA injection, whereas in WT animals the sensitivity to PTZ significantly decreased.

Intrinsic neuronal activation of RTK dependent signaling pathways appears to be sufficient to inhibit KA induced neuronal migration as demonstrated by the reduced GCD in Spry2/4^+/−^ mice. The broadening of the granule cell layer is observed ipsilaterally to the KA injection site only and does not involve neurogenesis, but requires translocation of the neuronal nucleus into an existing apical dendrite and is associated with lowered expression of the extracellular matrix protein Reelin (Bouilleret et al., 1999; Chai et al., 2013; Murphy and Danzer, 2011; Heinrich et al., 2006). Heinrich et al. also showed that neutralizing antibodies to Reelin induce GCD in control animals in vivo suggesting a function of Reelin in the stabilization of the compact granule cell layer in the adult dentate gyrus probably by inhibiting neuronal motility via modulating cofilin phosphorylation and, subsequently, the actin cytoskeleton (Chai et al., 2009). The dramatic loss of Reelin positive cells in the subgranular zone and hilus following KA injection was detected in both groups, WT and Spry2/4^+/−^ mice, to a similar extent suggesting that intrinsic neuronal mechanisms inducing migration, but not the synthesis or release of Reelin, are under the control of Spry proteins. Since there is cross‐talk between Ras/Raf/MAPK/ERK and Rho/ROCK/LIMK/cofilin signaling (Hensel et al., 2014), reduced Spry2/4 levels and enhanced ERK signaling may counteract the reduction in Reelin.

In conclusion, the present study for the first time provides evidence for a regulatory function of negative feedback inhibitors of RTK signaling in a mouse model of epileptogenesis resulting in neurodegeneration, neuronal migration and reactive astrogliosis. While astrogliosis is enhanced in Spry2/4^+/−^ mice, neuronal cell loss and GCD are clearly reduced indicating that Spry2/4 may serve as pharmacological targets in epilepsy or in other neurodegenerative diseases.
